# Trends in Resistance to Extended-Spectrum Cephalosporins and Carbapenems among *Escherichia coli* and *Klebsiella* spp. Isolates in a District in Western India during 2004–2014

**DOI:** 10.3390/ijerph15010155

**Published:** 2018-01-19

**Authors:** Ingvild Odsbu, Smita Khedkar, Frida Lind, Uday Khedkar, Sandeep S. Nerkar, Nicola Orsini, Ashok J. Tamhankar, Cecilia Stålsby Lundborg

**Affiliations:** 1 Department of Public Health Sciences, Karolinska Institutet, 17177 Stockholm, Sweden; adirf81@hotmail.com (F.L.); san.ner1978@gmail.com (S.S.N.); nicola.orsini@ki.se (N.O.); ejetee@gmail.com (A.J.T.); cecilia.stalsby.lundborg@ki.se (C.S.L.); 2 Bac-Test Laboratory, College Road, Nashik 422005, Maharashtra, India; mail@bactestlab.com (S.K.); udayk_nsk@sancharnet.in (U.K.); 3 Indian Initiative for Management of Antibiotic Resistance, Department of Environmental Medicine, R.D. Gardi Medical College, Ujjain 456006, India

**Keywords:** *Escherichia coli*, *Klebsiella*, India, ESBL, cephalosporins, carbapenems, antibiotic resistance, non-susceptible, antimicrobial susceptibility testing

## Abstract

Surveillance data on the level of resistant bacteria is needed to inform strategies to reduce the development and spread of antibiotic resistance. The aim of this study was to determine the non-susceptibility trends to extended-spectrum cephalosporins and carbapenems among *Escherichia coli* and *Klebsiella* spp. isolates from the district of Nashik in Western India during the period 2004–2014. Antibacterial susceptibility testing of clinical isolates was performed using Kirby-Bauer disc diffusion method to determine inhibitory zone diameters. The change in proportions of non-susceptible bacteria over calendar time was investigated with spline transformations in a logistic regression model. For the extended-spectrum cephalosporins, the proportions of non-susceptible *E. coli* and *Klebsiella* spp. isolates were above 78.4% and 84.9% throughout the study period, respectively. *E. coli* and *Klebsiella* spp. isolates exhibited carbapenem non-susceptibility levels as high as 76.9% and 84.1% respectively. The proportions of extended-spectrum betalactamase (ESBL)-producing isolates ranged from 38.3–85.9% in *E. coli* and from 45.1–93.1% in *Klebsiella* spp. Significantly higher proportions of non-susceptible and ESBL-producing isolates were found among isolates from inpatients compared to isolates from outpatients for both *E. coli* and *Klebsiella* spp. (*p* < 0.050). The high proportions of non-susceptible isolates observed show that there is great need to focus on optimal use of antibiotics to reduce the development of antibiotic resistance.

## 1. Introduction

The increase in antibiotic resistance has been acknowledged as one of the top three greatest threats to global health in our time [1]. In 2014, the World Health Organization (WHO) published a report on the global surveillance of antimicrobial resistance [2]. Mapping of the availability of national surveillance data on antimicrobial resistance worldwide showed large gaps, particularly in Africa and South and South-East Asia, which is of great concern. Without such data, we will not know the scope of the problem, we will not know where to put our efforts and we will not be able to detect new trends and threats. In the Global Action Plan on Antimicrobial Resistance (GAP-AMR, with special emphasis on antibiotic resistance) adopted by the World Health Assembly in May 2015, one of the objectives is to strengthen the knowledge and evidence base through surveillance and research [3]. As a response to the GAP-AMR, the WHO has launched an initiative called the Global Antimicrobial Resistance Surveillance System (GLASS) that aims to establish a global standardized approach to the collection of, analysis and sharing of data [4]. Setting up a national surveillance system can be a challenge to many countries, particularly in countries where health systems are weak. In addition to the costs of running a laboratory of high standards, there is a need for skilled personnel and standardized methods that allow for comparisons of resistance proportions between countries.

Since a national surveillance system is lacking in India, there is a great need for long-term studies on the proportions and trends in antibiotic resistance. Several long-term studies from India have found high antibiotic resistance proportions, but most of these studies are single-site studies or cover shorter periods of time [5,6,7,8,9,10,11]. Recently, a retrospective study over seven years from a private laboratory network in India concluded that there are high proportions of resistant bacterial strains among blood culture isolates from patients across India [12].

High proportions of resistant Gram-negative bacterial strains are of great concern since there are few treatment options left, especially for the extended-spectrum cephalosporin and carbapenem-resistant *E. coli* and *Klebsiella* spp. strains. In this study, we have examined the trends in resistance to extended-spectrum cephalosporins and carbapenems among clinical *E. coli* and *Klebsiella* spp. isolates in the district of Nashik in Western India over an 11-year period (2004–2014).

## 2. Materials and Methods

### 2.1. Study Setting

The data used in this study were primarily collected for antibacterial susceptibility testing of clinical bacterial isolates and secondarily for surveillance and research purposes. The antibacterial susceptibility testing was performed at the Bac-Test Laboratory, an International Organization for Standardization (ISO) certified laboratory that serves various hospitals, clinics and diagnostic laboratories in the district of Nashik [13], which belongs to the state of Maharashtra in the western part of India and has a population of more than 6.1 million and covers an area of 15,582 sq km [14].

### 2.2. Study Material

Information about the patient (gender), type of patient (in- or outpatient), type of specimen (urine, blood etc.), date of specimen collection, type of bacterial species and the results of the antibacterial susceptibility testing for every isolate were recorded in the WHONET software [15]. The WHONET is a free of charge software program developed by the WHO to enable collection of susceptibility data in resource-poor settings [15,16]. Information about several isolates (e.g., different year, different specimen types, and/or different species) may have been obtained from the same patient. The data were collected in the period from 1 January 2004 to 31 December 2014. All years contained data for every month of the year, except for 2010 which contained data for January only (hence the sample size is small (*n* < 30)). The data file for the rest of 2010 was corrupted at the laboratory and could not be revived. Type of patient (in- or outpatient) was only available for the period 2011–2014.

The data material used in this study was retrospective data from routine analysis of antibacterial susceptibilities. The data were anonymous since no patient identifiers were available, and it would therefore not be possible to obtain informed consent from the patients.

### 2.3. Laboratory Methods

Antibiotic susceptibility testing was performed using the Kirby-Bauer disk diffusion method (with disks from HiMedia Laboratories, Mumbai, India and Becton, Dickinson, Franklin Lakes, NJ, USA) according to the most updated Clinical and Laboratory Standards Institute (CLSI) guideline at the time of analysis [17,18]. The double disk synergy test was used to screen for extended-spectrum betalactamase (ESBL)-producing bacterial strains [19]. Some of the ESBL positive isolates may not possess antibacterial resistance genes, hence, the term “ESBL-positive” used here reflects an ESBL phenotype-positive isolate. The decision on which panel of antibiotics to test for a particular isolate was based on type of bacterial strain, type of specimen (different infections require different panels of antibiotics to be tested), and sometimes specific requirements of the clinicians.

### 2.4. Statistical Analyses

For analyses purposes we interpreted the recorded inhibitory zone diameters according to the clinical zone diameter breakpoints provided by the CLSI guideline used at the time of the antibacterial susceptibility testing [19]. Susceptible bacterial isolates were classified as “susceptible”, whereas intermediate susceptible and resistant bacterial isolates were classified as “non-susceptible”. The antibacterial categories included in the study were extended-spectrum cephalosporins (cefepime, cefixime, cefotaxime, cefpodoxime, and ceftazidime) and carbapenems (meropenem and imipenem). Non-susceptibility to an antibacterial category was defined as being non-susceptible to at least one antibacterial agent in an antibacterial category [20]. Changes in the CLSI guidelines for clinical zone diameter breakpoints for cefotaxime and ceftazidime in 2010, meropenem and imipenem in 2011, and cefepime in 2014 were accounted for when classifying bacterial isolates as “susceptible” or “non-susceptible”.

Proportions of non-susceptible bacterial isolates (number of non-susceptible isolates as a proportion of total isolates tested) were calculated from the antibacterial susceptibility data. Calendar time (year) was flexible modelled as a continuous predictor using regression splines in two ways. First, a principled approach known as multivariable regression spline approach (MVRS) [21], was used to flexibly smooth the trajectory over time without imposing any constraints on the functional relation. The MVRS selected the model that had the best fit of the data using the smallest number of parameters. Second, a visual inspection of the calculated proportions of non-susceptible isolates over time was done by the authors and along with the predicted smooth trends these two approaches provided the basis to identify the most likely point in time where the outcome may have changed (knot). For its simplicity, we approximated the trajectories of proportions of non-susceptible bacterial isolates over calendar time with two linear trends, one before the selected knot and one after the selected knot. Odds ratios (ORs) of non-susceptible bacterial isolates associated with 1-year increment before and after the knot were estimated along with 95% confidence intervals using piece-wise linear splines. Calculated proportions of non-susceptible isolates were compared between inpatients and outpatients using Chi square test. A two-sided *p*-value < 0.05 was considered statistically significant.

Statistical analyses were performed using Stata version 14 (Stata Corp, TX, USA).

## 3. Results

### 3.1. Bacterial Isolates

The data set contained information about 9813 clinical isolates collected during the period 2004–2014 out of which 5863 isolates were identified as *E. coli*, and 3950 isolates were identified as *Klebsiella* spp. The most common specimen types for *E. coli* isolates were urine (56.6%), stool (18.5%) and pus (12%). For *Klebsiella* spp. isolates the most common specimen types were urine (31.1%), from the respiratory tract (21.0%) and pus (20.6%). The proportions of isolates from blood were 2.3% and 10.8% for *E. coli* and *Klebsiella* spp., respectively. The isolates originated to a slightly higher extent from males (58.2%) compared to females (41.8%) for *Klebsiella* spp., while no sex differences were observed for *E. coli* isolates. About 60% of the *E. coli* isolates and 70% of the *Klebsiella* spp. isolates came from inpatients, and these proportions were consistent for every year in the period during which this information was available (2011–2014).

### 3.2. Trends in Non-Susceptible E. coli and Klebsiella spp. Isolates during the Period 2004–2014

**Figure 1:**
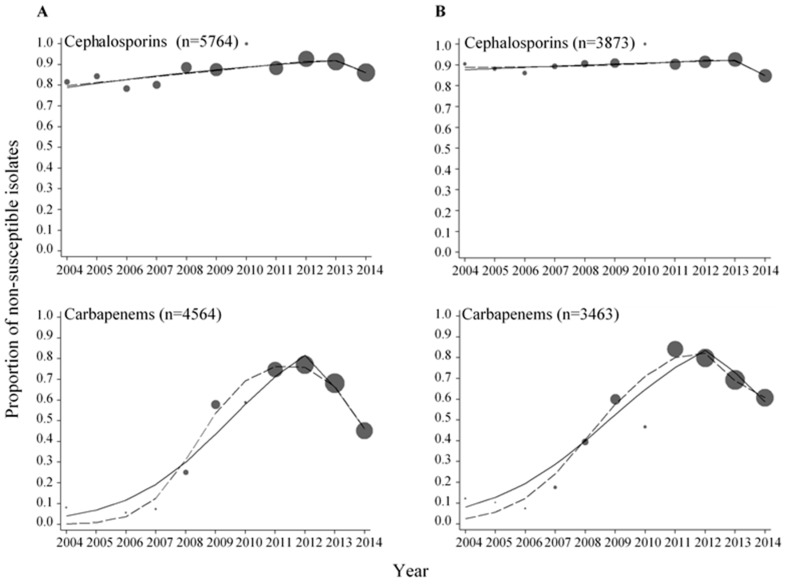
Proportions of *E. coli* (**A**) and *Klebsiella* spp. (**B**) isolates non-susceptible to cephalosporins and carbapenems. The data were fitted with a piece-wise linear logistic regression model (solid line) and multivariable regression spline procedure (MVRS) (dashed line). Each bubble shows the proportion of non-susceptible isolates to the specific antibacterial category for the years 2004–2014. The size of the bubbles represents the number of isolates tested per year relative to the total number of isolates tested (*n*) (see Table A1). *n* = total number of isolates tested for the specific antibacterial category in the whole period.

For the *E. coli* isolates, 98.3% (*n* = 5764) were tested for susceptibility to extended-spectrum cephalosporins and 77.8% (*n* = 4564) were tested for susceptibility to carbapenems. For the *Klebsiella* spp. isolates, 98.1% (*n* = 3873) were tested for susceptibility to extended-spectrum cephalosporins and 87.7% (*n* = 3463) were tested for susceptibility to carbapenems. High proportions of non-susceptible *E. coli* and *Klebsiella* spp. isolates were found for the extended-spectrum cephalosporins throughout the period, ranging from 78.4 to 100% and from 84.9 to 100%, respectively (Figure 1 and Table A1).

We next analyzed trends in proportions of non-susceptible isolates using splines of calendar time. The year 2013 was identified as the year where a change in the proportions of extended-spectrum cephalosporin non-susceptible *E. coli* and *Klebsiella* spp. isolates most likely occurred (knot year) (Table 1). Per every year increment in the period 2004–2013 there was a 13% increase in the odds of an *E. coli* isolate being non-susceptible to extended-spectrum cephalosporins, whereas in the period 2013–2014 there was a 52% decrease in the odds of an *E. coli* isolate being non-susceptible. For *Klebsiella* spp., there was a 6% increase in the odds of an isolate being non-susceptible to extended-spectrum cephalosporins per every year increment in the period 2004–2013, whereas in the period 2013–2014 there was a 56% decrease in the odds of an isolate being non-susceptible. Hence, for both *E. coli* and *Klebsiella* spp. there was an increasing trend in the proportions of non-susceptible isolates before 2013 and a decreasing trend in the proportions of non-susceptible isolates after 2013. For the carbapenems, the proportions of non-susceptible isolates ranged from 0 to 76.9% in *E. coli* and from 7.4 to 84.1% in *Klebsiella* spp., respectively (Figure 1 and Table A1). Increasing trends in the proportions of carbapenem non-susceptible *E. coli* and *Klebsiella* spp. isolates were found before 2012 (knot year) and decreasing trends were found after 2012 (Table 1).

**Figure A1:**
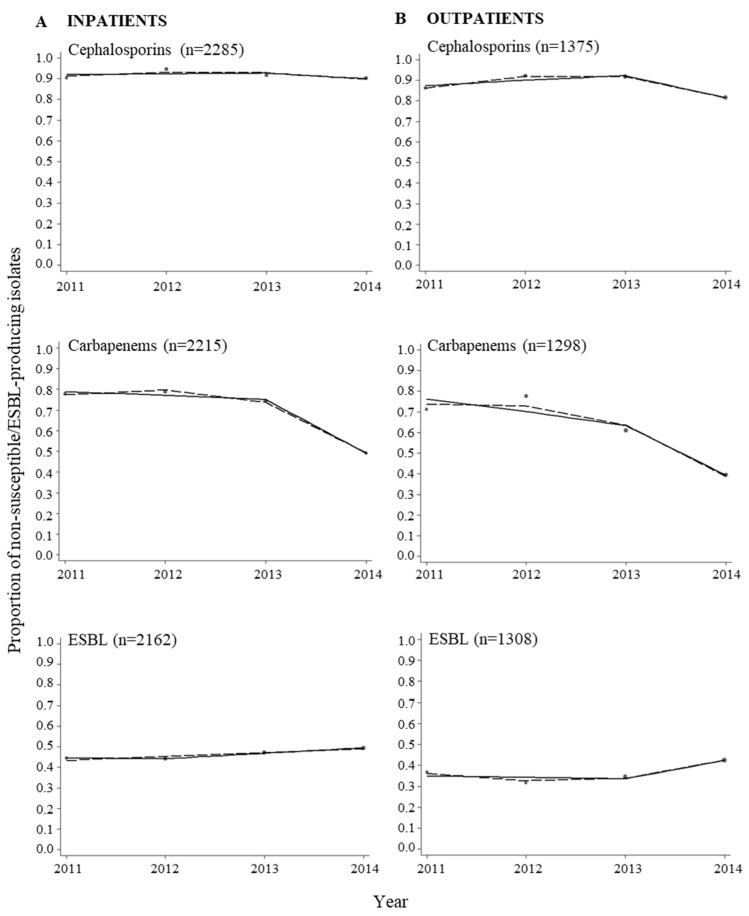
Proportions of *E. coli* isolates non-susceptible to cephalosporins and carbapenems, and extended-spectrum betalactamase (ESBL)-producing isolates from inpatients (**A**) and outpatients (**B**). The data were fitted with a piece-wise linear logistic regression model (solid line) and multivariable regression spline procedure (MVRS) (dashed line). Each bubble shows the proportion of isolates non-susceptible to the specific antibacterial category or the proportion of ESBL-producing isolates for the years 2011–2014. The size of the bubbles represents the number of isolates tested per year relative to the total number of isolates tested (*n*) (see Table A2). *n* = total number of isolates tested for the specific antibacterial category or ESBL-production in the whole period.

**Figure A2:**
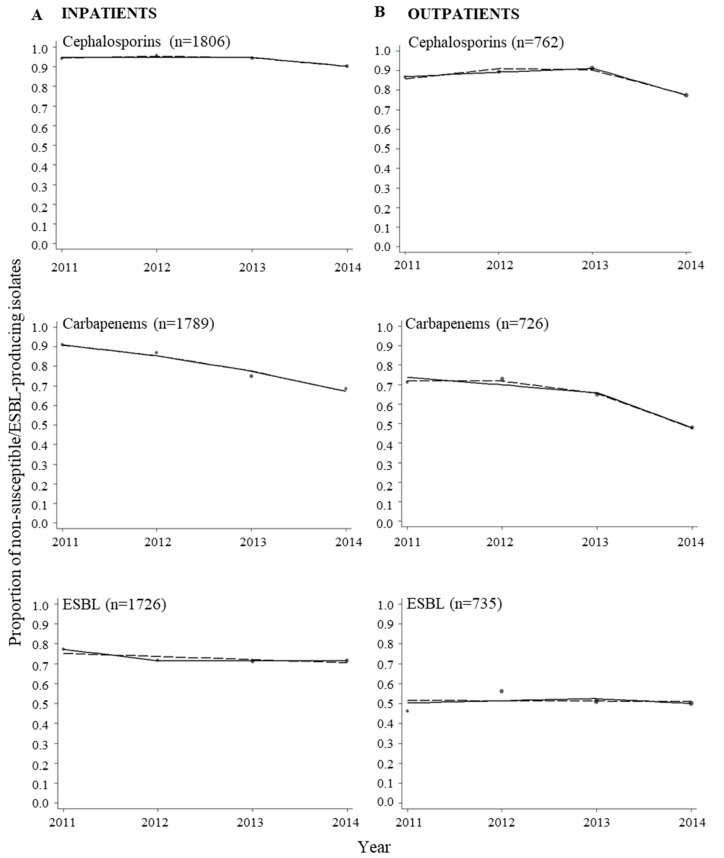
Proportions of *Klebsiella* spp. isolates non-susceptible to cephalosporins and carbapenems, and extended-spectrum betalactamase (ESBL)-producing isolates from inpatients (**A**) and outpatients (**B**). The data were fitted with a piece-wise linear logistic regression model (solid line) and multivariable regression spline procedure (MVRS) (dashed line). Each bubble shows the proportion of isolates non-susceptible to the specific antibacterial category or the proportion of ESBL-producing isolates for the years 2011–2014. The size of the bubbles represents the number of isolates tested per year relative to the total number of isolates tested (*n*) (see Table A2). *n* = total number of isolates tested for the specific antibacterial category or ESBL-production in the whole period.

Significantly higher proportions of extended-spectrum cephalosporin and carbapenem non-susceptible isolates were found in inpatients when comparing non-susceptibility rates among isolates from inpatients and outpatients for both *E. coli* and *Klebsiella* spp. (*p* < 0.050, Table 2). When comparing the trends in proportions of non-susceptible *E. coli* and *Klebsiella* spp. isolates during the period 2011–2014, there were no major differences observed among isolates from inpatients compared to isolates from outpatients (Figure A1 and Figure A2, Table A2 and Table A3). Although some statistically significant changes were observed (Table A3), particularly among isolates from outpatients, a longer time period than four years would be required to allow for more robust analyses of the trends.

When comparing non-susceptibility rates among urinary samples (the most common specimen type) and all other specimen types, significantly lower proportions of extended-spectrum cephalosporin and carbapenem non-susceptible isolates were found among urinary samples for both *E. coli* and *Klebsiella* spp. (*p* < 0.050, Table A4).

Significantly higher proportions of extended-spectrum cephalosporin and carbapenem non-susceptible isolates were found among males compared to females (*p* < 0.050, Table A5).

### 3.3. Trends in ESBL-Producing E. coli and Klebsiella spp. Isolates during the Period 2006–2014

**Figure 2:**
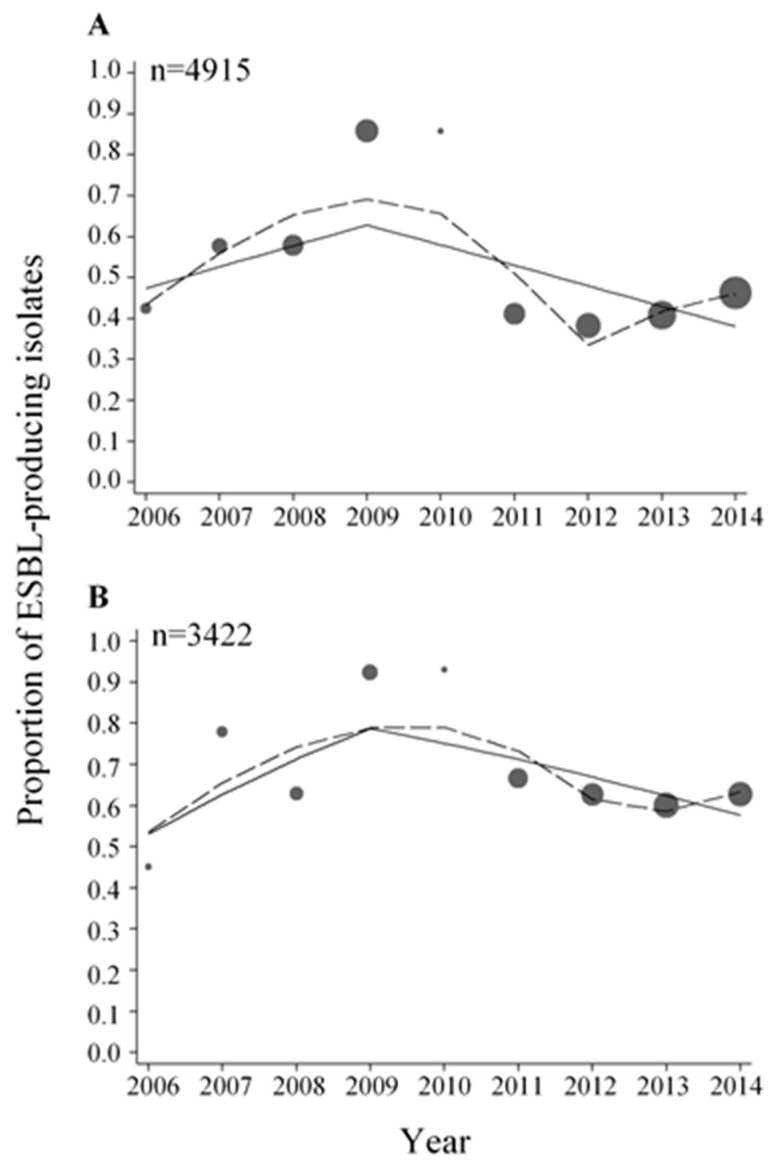
Proportions of ESBL-producing *E. coli* (**A**) and *Klebsiella* spp. (**B**) isolates. The data were fitted with a piece-wise linear logistic regression model (solid line) and multivariable regression spline procedure (MVRS) (dashed line). Each bubble shows the proportion of ESBL-producing *E. coli* and *Klebsiella* spp. isolates for the years 2006–2014. The size of the bubbles represents the number of isolates tested per year relative to the total number of isolates tested (*n*) (see Table A1). *n* = total number of isolates tested for ESBL in the whole period.

Of the 5589 *E. coli* isolates analysed during the period 2006–2014, 4915 isolates (87.9%) were tested for ESBL. The proportions of ESBL-producing *E. coli* ranged from 38.3 to 85.9% (Figure 2A and Table A1) during these years. Per every year increment in the period 2006–2009, there was a 23% increase in the odds of *E. coli* isolates being ESBL-positive, whereas in the period 2009–2014 there was a 34% decrease in the odds of *E. coli* isolates being ESBL-positive (Table 1). For *Klebsiella* spp., 3422 out of 3810 isolates (89.8%) were tested for ESBL in the period 2006–2014. The proportions of ESBL-producing *Klebsiella* spp. ranged from 45.1 to 93.1% (Figure 2B and Table A1). As for ESBL-producing *E. coli* isolates, an increasing trend in the proportions of ESBL-producing *Klebsiella* spp. was observed before 2009 and a decreasing trend was observed after 2009 (Table 1).

Significantly higher proportions of ESBL-producing isolates were found in inpatients when comparing proportions of ESBL-positive isolates from inpatients and outpatients for both *E. coli* and *Klebsiella* spp. (*p* < 0.050, Table 2). No major differences in trends were observed among isolates from inpatients compared to isolates from outpatients in the period 2011–2014 (Figure A1 and Figure A2, Table A2 and Table A3).

When comparing the proportions of ESBL-producing isolates from urinary samples and all other specimen types, significantly lower proportions of ESBL-producing isolates were found among urinary samples for both *E. coli* and *Klebsiella* spp. (*p* < 0.050, Table A4).

Significantly higher proportions of ESBL-producing isolates were found among males compared to females (*p* < 0.050, Table A5). 

## 4. Discussion

We have examined data on antibacterial susceptibility over an 11-year period among *E. coli* and *Klebsiella* spp. isolates from community- and hospital-acquired infections in the district of Nashik in Western India. High proportions of extended-spectrum cephalosporin and carbapenem non-susceptible isolates, as well as high proportions of ESBL-producing isolates were found for both *E. coli* and *Klebsiella* spp. In our data, we observed that the proportions of non-susceptible bacterial isolates did not show a steady increase or decrease over time. Rather, the proportions fluctuated over time. This has also been observed in other long-term studies of trends in antibiotic resistance [5,22,23,24] and might reflect differences in the annual burden of infections, changes over time in the choice of empiric drugs for treatment of infections and changes in the study population.

We found that the proportions of non-susceptible isolates were higher in inpatients compared to outpatients. Higher prevalence of resistance among bacterial isolates from inpatients compared to outpatients has also been found in other studies [25,26]. Higher prevalence of resistance was also found among bacterial isolates from males compared to females. The differences observed between males and females could be due to differences in exposure to bacterial pathogens and/or antibiotics. Differences in the proportions of non-susceptible isolates were also found when comparing urinary samples and all other specimen types for both *E. coli* and *Klebsiella* spp. Lower proportions of non-susceptible isolates from urinary samples might reflect that cephalosporins and carbapenems are not recommended as first line treatment of urinary tract infections. Despite lower proportions of non-susceptible isolates among urinary samples compared to other specimen types, the proportions are alarmingly high. Analysis of nitrofurantoin non-susceptible *E. coli* isolates during the period 2004–2014 revealed that the non-susceptibility proportions ranged from 4.9–39.8% (data not shown). The lower prevalence of resistant *E. coli* isolates observed for nitrofurantoin, one of the drugs recommended as first line treatment of urinary tract infections [27], compared to cephalosporins and carbapenems might indicate irrational use of antibiotics to treat urinary tract infections.

To analyse the trends in proportions of non-susceptible isolates, we modelled calendar time using two different approaches, namely a multivariable regression spline model to flexibly model time without constraints and a piece-wise linear spline to summarize the overall change of non-susceptibility over time. Using these approaches, we were able to detect a point in time where a change in the proportions of non-susceptible isolates most probably occurred. In some cases, the MVRS approach indicated complicated trends with probably more than a single point of change in slope. For example, for ESBL-producing *E. coli* and *Klebsiella* spp. (Figure 2), the year 2009 was chosen as the knot year (Table 1) resulting in an increasing trend in ESBL-producing isolates before 2009 and a decreasing trend after 2009. From the graph showing the predicted proportions from the MVRS approach, an increasing trend was predicted up to the year 2009, followed by a decreasing trend to the year 2012 in *E. coli* and 2012–2013 in *Klebsiella* spp., and then an increasing trend from 2012 and 2013, respectively. It would be possible to choose more than one knot to detect more than one change in the trend, but we wanted to grasp the major changes and therefore chose to use one knot in our analyses.

The years 2009, 2012, and 2013 were identified as the years where a change in the odds of bacterial isolates being non-susceptible or ESBL-producing most likely occurred (Table 1). In the period following these years, decreasing trends in non-susceptible and ESBL-producing isolates were detected for both *E. coli* and *Klebsiella* spp. Since we did not have data on antibiotic consumption, neither from the individual patient nor at the aggregated level from the district of Nashik, we can only speculate about the reason for this observation. The WHO selected antimicrobial resistance as theme for World Health Day 2011 [28], and in accordance to this activity directed towards physicians were conducted in the district of Nashik in order to promote rational use of antibiotics. This was done through the Indian Initiative of Management of Antibiotic Resistance (IIMAR) [29]. IIMAR activities were continued further in the program of the Antibiotic Stewardship Network in India [30] in 2013–2014 in the Nashik district. These activities were focusing on proper use of antibiotics, particularly prescription of last resort antibiotics, like carbapenems, to treat infections caused by Gram-negative bacteria (unpublished information). Many factors influence prescribing decisions, and raising awareness and knowledge among physicians have been shown to be important to improve the prescribing of antibiotics [31,32]. The decreasing trends in non-susceptible and ESBL-producing isolates observed in the last part of the study period might be attributed to these activities, and also to the increased focus on antibiotic resistance in general, both in India and the rest of the world [2,27,33].

High proportions of extended-spectrum cephalosporin and carbapenem non-susceptible isolates were observed in this study. For the extended-spectrum cephalosporins, the proportions of non-susceptible *E. coli* and *Klebsiella* spp. isolates were above 78.4% and 84.9%, respectively, throughout the period 2004–2014. For the carbapenems, there were much more variations in the proportions of non-susceptible *E. coli* and *Klebsiella* spp. isolates. Most samples were collected from 2008 and onwards which reflects the increasing use of carbapenems in the recent years due to increased availability and reduced cost of these drugs (unpublished information). Similar proportions of extended-spectrum cephalosporin and carbapenem non-susceptible isolates have been reported in other studies from India [10,12,34]. The high proportions of inpatients (or only inpatients) in the study populations can explain the high proportions of resistance observed in these studies. A study on the proportions of resistance in bacteria isolated from urine in individuals in the community in Northern India revealed low proportions for extended-spectrum cephalosporins, and no resistance towards carbapenems [35].

The proportions of extended-spectrum cephalosporin and carbapenem non-susceptible isolates found in this study are alarmingly high. In Europe, where most countries have national surveillance systems, the population-weighted mean resistance percentages in 2014 were 12% for extended-spectrum cephalosporins and <0.1% for carbapenems among invasive *E. coli* isolates. Among invasive *Klebsiella* spp. isolates the percentages were 28% and 7.3%, respectively [36]. Like in India there are variations in the proportions of resistance reported by various regions of Europe, with highest resistance proportions reported from southern and south-eastern Europe.

Of particular concern are the high proportions of ESBL-producing isolates found both in *E. coli* and *Klebsiella* spp. High proportions of ESBL-producing bacteria and a rapid increase in recent years have also been observed in other studies from India [5,6,7,37,38]. The high proportions found are worrisome since there are few treatment options left to treat infections caused by these bacteria, particularly if there is also resistance to carbapenems.

### Strengths and Limitations

The data analysed in this study are unique, since longitudinal surveillance data from India are scarce. The data were collected over an 11-year period and analysed at the same laboratory. Although there could be changes in staff and laboratory practices over time, the fact that the same laboratory performed all antibacterial susceptibility testing minimizes the risk for bias due to methodological issues. The availability of data on type of patient made it possible to distinguish between inpatients and outpatients in the period 2011–2014. The use of regression splines instead of using the overall linear trend allowed for more robust analysis of the trends in non-susceptibility over time. A limitation to our study is that a large proportion of the data was collected during the second half of the study period, making the estimates for the first half of the period less robust compared to estimates from the second half of the study period. No patient identifiers were available in the data set meaning that the number of patients included in the study was not known.

## 5. Conclusions

This study highlights the high proportion of antibiotic resistance in India and the need for a national surveillance system. In addition, there is great need to focus on optimal use of antibiotics as well as infection prevention and control measures to combat the emerging threat of antibiotic resistance. Although decreasing trends in antibiotic resistance among *E. coli* and *Klebsiella* spp. isolates were observed in recent years, it cannot be ruled out that the trends might increase again which shows the importance of longitudinal surveillance data to detect changes in antibiotic resistance trends.

## Figures and Tables

**Table 1:** Odds ratios (OR) with 95% confidence intervals (CI) of non-susceptible isolates for every year increase in the period 2004–2014 (2006–2014 for ESBL-producing isolates) from a piece-wise logistic regression model.

Organism	Antibacterial Category/ESBL	Linear Trend				
		Before the Knot		Knot Year	After the Knot	
		OR	95% CI		OR	95% CI
*E. coli*	Cephalosporins	1.13	1.09–1.17	2013	0.48	0.38–0.61
	Carbapenems	1.80	1.71–1.89	2012	0.24	0.21–0.27
	ESBL-positive	1.23	1.11–1.37	2009	0.66	0.58–0.76
*Klebsiella* spp.	Cephalosporins	1.06	1.01–1.11	2013	0.44	0.33–0.59
	Carbapenems	1.66	1.57–1.75	2012	0.32	0.28–0.37
	ESBL-positive	1.48	1.29–1.71	2009	0.55	0.46–0.66

ESBL: extended-spectrum betalactamase.

**Table 2:** Proportions (%) of non-susceptible or ESBL-producing isolates from inpatients and outpatients according to antibacterial category and ESBL production in the period 2011–2014.

Antibacterial Category/ESBL	*E. coli*			*Klebsiella* spp.		
	Inpatients	Outpatients	*p* ^1^	Inpatients	Outpatients	*p* ^1^
Cephalosporins	91.6	87.5	<0.001	93.4	85.8	<0.001
Carbapenems	67.9	58.4	<0.001	78.8	62.0	<0.001
ESBL-positive	46.7	36.9	<0.001	72.5	51.2	<0.001

^1^ *p* < 0.050 (Chi square test, 2-tailed) indicates statistical significance of the differences in non-susceptibility rates. ESBL: extended-spectrum betalactamase.

## Appendix A

**Table A1:** Proportions (%) of non-susceptible or ESBL-producing *E. coli* and *Klebsiella* spp. isolates and total number (*n*) of isolates tested according to antibacterial category, ESBL production and year.

Organism	2004		2005		2006		2007		2008		2009		2010		2011		2012		2013		2014	
	%	*n*	%	*n*	%	*n*	%	*n*	%	*n*	%	*n*	%	*n*	%	*n*	%	*n*	%	*n*	%	*n*
*E. coli*																						
Cephalosporins	81.5	119	84.2	133	78.4	171	80.1	246	88.6	429	87.4	650	100	28	88.2	721	92.7	904	91.4	1115	86.1	1248
Carbapenems	8.1	62	0	38	5.6	90	7.3	109	25.1	195	57.8	263	58.8	17	74.6	627	76.9	849	68.1	1086	45.1	1228
ESBL-positive	N/A	N/A	N/A	N/A	42.4	151	57.7	222	57.9	411	85.9	325	85.7	21	41.1	626	38.3	874	40.8	1067	46.2	1218
*Klebsiella* spp.																						
Cephalosporins	90.4	52	88.2	76	86	93	89.3	140	90.4	230	90.8	381	100	34	90.4	518	91.5	649	92.6	881	84.9	819
Carbapenems	12.1	33	10.3	29	7.4	54	17.5	97	39.4	170	60	270	46.7	30	84.1	483	79.8	639	69.2	858	60.6	800
ESBL-positive	N/A	N/A	N/A	N/A	45.1	82	78.0	127	63	227	92.3	220	93.1	29	66.7	468	62.7	641	60	838	62.7	790

ESBL: extended-spectrum betalactamase. *n* = total number of isolates tested for the specific antibacterial category or ESBL-production in the specific year.

**Table A2:** Proportions (%) of non-susceptible or ESBL-producing *E. coli* and *Klebsiella* spp. isolates according to antibacterial category, ESBL production and year.

Patient Type	Organism	Antibacterial Category/ESBL	2011		2012		2013		2014	
			%	*n* ^1^	%	*n* ^1^	%	*n* ^1^	%	*n* ^1^
Inpatient	*E. coli*	Cephalosporins	90.4	428	94.7	527	91.6	618	90.0	712
		Carbapenems	77.7	386	78.9	512	74.3	610	49.1	707
		ESBL-positive	44.4	369	44.0	511	47.2	589	49.4	693
	*Klebsiella* spp.	Cephalosporins	94.2	345	95.6	410	94.3	527	90.3	524
		Carbapenems	91.0	333	87.0	414	75.0	519	68.5	523
		ESBL-positive	77.2	316	71.7	406	71.1	501	71.6	503
Outpatient	*E. coli*	Cephalosporins	86.1	223	92.1	280	91.6	417	81.5	455
		Carbapenems	71.2	191	77.6	259	60.9	404	39.4	444
		ESBL-positive	36.8	193	31.5	267	34.5	400	42.4	448
	*Klebsiella* spp.	Cephalosporins	86.7	98	89.4	160	91.2	260	77.5	244
		Carbapenems	71.1	90	72.9	155	65.0	251	47.8	230
		ESBL-positive	46.2	91	56.1	157	51.0	249	50.0	238

^1^ *n* = total number of isolates tested. ESBL: extended-spectrum betalactamase.

**Table A3:** Odds ratios (OR) and 95% confidence intervals (CI) of non-susceptible isolates from inpatients and outpatients for every year increment during the years 2011–2014 from a piece-wise logistic regression model.

Patient Type	Organism	Antibacterial Category/ESBL	Linear Trend				
			Before the Knot		Knot Year	After the Knot	
			OR	95% CI		OR	95% CI
Inpatient	*E. coli*	Cephalosporins	1.05	0.84–1.32	2013	0.69	0.40–1.17
		Carbapenems	0.90	0.77–1.04	2013	0.36	0.26–0.50
		ESBL-positive	0.99	0.77–1.28	2012	1.12	0.81–1.56
	*Klebsiella* spp.	Cephalosporins	0.99	0.73–1.34	2013	0.53	0.27–1.04
		Carbapenems	0.58	0.37–0.90	2012	1.03	0.60–1.75
		ESBL-positive	0.74	0.53–1.02	2012	1.35	0.89–2.05
Outpatient	*E. coli*	Cephalosporins	1.32	1.01–1.72	2013	0.28	0.15–0.52
		Carbapenems	0.73	0.61–0.88	2013	0.52	0.35–0.76
		ESBL-positive	0.97	0.81–1.16	2013	1.50	1.01–2.22
	*Klebsiella* spp.	Cephalosporins	1.25	0.88–1.79	2013	0.26	0.12–0.57
		Carbapenems	0.83	0.64–1.07	2013	0.57	0.34–0.97
		ESBL-positive	1.05	0.83–1.32	2013	0.86	0.53–1.42

ESBL: extended-spectrum betalactamase.

**Table A4:** Proportions (%) of non-susceptible isolates according to specimen type and antibacterial category during the years 2004–2014, and proportions (%) of ESBL-producing isolates according to specimen type during the years 2006–2014.

Antibacterial Category/ESBL	*E. coli*			*Klebsiella* spp.		
	Urinary	Other	*p* ^a^	Urinary	Other	*p* ^a^
Cephalosporins	85.2	92.0	<0.001	86.8	91.2	<0.001
Carbapenems	54.8	61.7	<0.001	61.8	67.0	0.003
ESBL-positive	42.3	53.6	<0.001	58.6	67.7	<0.001

^a^ *p* < 0.050 (Chi square test, 2-tailed) indicates statistical significance of the differences in non-susceptibility rates. ESBL: extended-spectrum betalactamase.

**Table A5:** Proportions (%) of non-susceptible or ESBL-producing isolates according to gender and antibacterial category in the period 2004–2014, and according to gender and ESBL production in the period 2006–2014.

Antibacterial Category/ESBL	*E. coli*			*Klebsiella* spp.		
	Males	Females	*p* ^a^	Males	Females	*p* ^a^
Cephalosporins	91.8	84.7	<0.001	91.3	85.7	<0.001
Carbapenems	60.6	54.9	<0.001	69.4	58.1	<0.001
ESBL-positive	50.6	43.3	<0.001	68.6	54.9	<0.001

^a^ *p* < 0.050 (Chi square test, 2-tailed) indicates statistical significance of the differences in non-susceptibility rates. ESBL: extended-spectrum betalactamase.
